# Monoenergetic 290 MeV/n carbon-ion beam biological lethal dose distribution surrounding the Bragg peak

**DOI:** 10.1038/s41598-019-42600-4

**Published:** 2019-04-16

**Authors:** Dylan J. Buglewicz, Austin B. Banks, Hirokazu Hirakawa, Akira Fujimori, Takamitsu A. Kato

**Affiliations:** 1 Department of Environmental & Radiological Health Sciences, Colorado State University, Colorado, 80523 USA; 2 Department of Basic Medical Sciences for Radiation Damages, National Institute of Radiological Sciences, Chiba, 263-8555 Japan

## Abstract

The sharp high dose Bragg peak of a carbon-ion beam helps it to deliver the highest dosage to the malignant cells while leaving the normal cells relatively unharmed. However, the precise range in which it distributes dosages that significantly induce cell death or genotoxicity surrounding its Bragg peak remains unclear. To evaluate biological effects of carbon-ion radiation through entrance to post Bragg peak in a single biological system, CHO and xrs5 cells were cultured in T-175 cell culture flasks and irradiated with 290 MeV/n monoenergetic carbon-ions with initial dosages upon entrance to the flask of 1, 2, or 3 Gy for cell survival assays or 1 Gy for cytokinesis block micronuclei assays. Under all initial dosages, the biological Bragg peak and the highest micronuclei formation was observed at the depth of 14.5 cm. Moreover, as the initial dosage increased the range displaying a significant decrease in survival fraction increased as well (*P* < *0*.*0001*). Intriguingly from 1 Gy to 3 Gy, we observed a significant increase in reappearance of colony formation depth (*P* < *0*.*05*), possibly indicating the nuclear fragmentation lethality potential of the carbon-ion. By means of our single system approach, we can achieve a more comprehensive understanding of biological effects surrounding of carbon-ions Bragg peak.

## Introduction

Emerging advantages of carbon-ion radiotherapy (CIRT) have led to an increase of these facilities worldwide, with 12 operational facilities to date and 25,702 patients treated per end of 2017^1–3^. CIRT has been very successful in treating solid cancers, in which cell death occurs predominantly resulting from aberrant mitotic events known as mitotic catastrophe^4,5^, due to carbon-ion’s excellent physical dose-distribution and deposition around the Bragg peak. This provides it with a selective advantage, as it allows for the preservation of adjacent organs and their functions, which would be very difficult to achieve through highly invasive surgical procedures^5^. Furthermore, as location and radioresistance of certain tumors has also created implications in their treatment with other conventional low-LET (Linear Energy Transfer) radiotherapy modalities, high-LET carbon-ions have shown promising results in effectively treating these tumors. Most notably these include: skull base tumors, lung cancer, head and neck cancer, prostate cancer, liver cancer, pancreatic cancer, uterine cervical cancer, pelvic recurrences of rectal cancer, bone and soft tissue sarcoma^6–8^.

CIRT is a form of particle radiotherapy which gradually deposits an increasing amount of energy in the cells of tissues along its path as it passes through^9,10^. The depth at which the majority of the particle beam’s energy is deposited along the beam path is known as the “Bragg peak,” typically found at the end of the beams range^11^. This Bragg peak region has the maximum energy deposition of high-LET radiation^12^. In clinics, expanded Bragg peak, Spread out Bragg peak (SOBP) is used to cover the entire tumor volume^13,14^. Beyond the Bragg peak, there is a rapid falloff of the dose, allowing for sparing of the normal tissues at the distal edge of the tumor^10,15^. Therefore, the sharp high dose, as well as, the high-LET properties of the carbon-ion beam allow for maximum biological effectiveness at the Bragg peak and aid CIRT in achieving the fundamental principle of radiotherapy, to ensure precise localization of dose distribution to the target tumor while minimizing dose/damage to the surrounding normal tissues^15–17^.

However, previous studies have revealed that nuclear fragments of the carbon-ions, known as the carbon-ion fragmentation tail, can continue travelling at nearly the same velocity and direction beyond the carbon-ion Bragg peak^18,19^. Therefore, because of the carbon-ion sharp narrow Bragg peak and dose from the nuclear fragmentation, the extent of cell damage surrounding the Bragg peak remains unclear and must be addressed to further understand the precision of carbon-ion biological dose distribution and help define the extent of unwanted cellular damage surrounding its Bragg peak. These results will aid medical professionals utilizing CIRT to specify the dose distribution to the target tumor. In the present study, we aim to fill a gap in current research involving the range in which the monoenergetic 290 MeV/n carbon-ion beam distributes biologically lethal dosages. Our development of an *in-vitro* cell survival assay variant technique has enabled us with the ability to investigate biological effects near the Bragg peak precisely.

## Results

### Dose distribution and identification of biological Bragg peak

**Figure 1:**
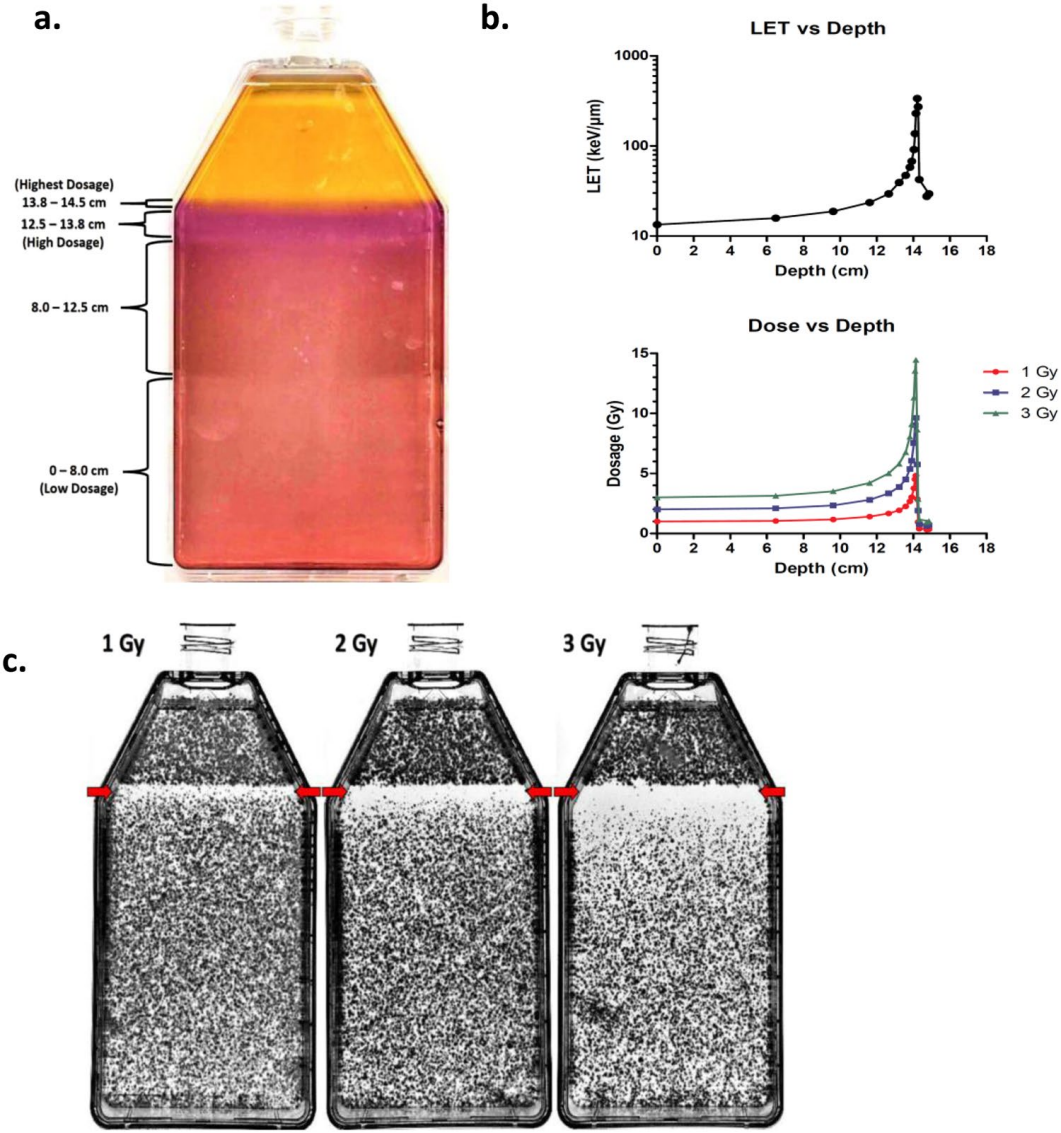
Dose distribution and biological Bragg peak depth. (**a**) Fricke gel dosimetry visual representation of carbon-ion beam depth vs dose distribution in our T-175 cell culture flask. Fricke gel indicated the highest dosage, therefore the Bragg peak, was located between the depths of 13.8 and 14.5 cm. (**b**) Graphs depicting LET vs depth (top) and dosage vs depth (bottom) from the calculated values of LET and dosage at specific depths provided by faculty at NIRS. (**c**) Images of cell survival assay T-175 cell culture flasks following carbon-ion beam irradiation of 1, 2 or 3 Gy dosages. Flasks portrayed the least number of colonies under all initial radiation conditions at the depth of 14.5 cm, representative of our biological Bragg peak depth. Red arrow: indicates biological Bragg peak.

Depth vs dose distribution across our T-175 flask was visually represented utilizing a Fricke gel dosimeter, as previous studies have reported ferrous xylenol gels can be used as dosimeters and can therefore provide a quality method for detecting dose distribution^20,21^. Irradiation oxidizes Fe^2+^ to Fe^3+^ which changes the color of the originally yellow gel to various orange-color combinations depending on the dosage at that specific depth. Increasing dosages indicated via color change in our flask were as follows: orange-pink, indicating low dosage was observed from beam entry (non-capped end) to 8.0 cm; orange-light red, indicating an increase in dose was observed between 8.0 and 12.5 cm; orange-purple, indicating a further increase in dosage was observed between 12.5 and 13.8 cm; orange-dark red, indicating the highest dose values was observed between 13.8 and 14.5 cm. Beyond 14.5 cm, the Fricke gel remained its originally yellow color, indicating a steep drop off of dosage shortly following this depth. These results indicated that the biological Bragg peak should be within the ranges of depths of 13.8 and 14.5 cm (Fig. 1a). Using the calculated data provided by faculty at the National Institute of Radiological Sciences (NIRS) LET vs depth and dose vs depth graphs were created (Fig. 1b). These graphs were consistent with our Fricke gel results depicting the highest LET and dose being between 13.8 and 14.5 cm. In addition, a previous study conducted by Weyrather *et al*. reported that the maximum RBE was found at LET between 150 and 200 keV/μm which is consistent with the LET values between these depths^22^ (Fig. 1b). Furthermore, under all initial irradiation conditions of 1, 2 or 3 Gy, the depth of 14.5 cm portrayed the smallest observable number of colonies (Fig. 1c). Therefore, we determined this depth to be the biological Bragg peak depth. Moreover, as the initial dosage increased, the range in which there was a dramatic decrease in colony formation surrounding our biological Bragg peak was observed to also increase. Thus, we referred to this range as the lethal dosage range.

### Proximal and distal ends of biological lethal dose range

For each of our experimental initial beam entry irradiation dosages, all evaluated depths were compared to our first evaluated depth at 2.0 cm from the carbon-ion beam entry into our flask, allowing us to observe any significant decreases or increases in survival fractions at different depths of the carbon-ion beam. We observed a decrease in survival at our first evaluated depth with an increase of initial beam entry irradiation dosage. This observation was consistent with previous studies conducted by M P Carante *et al*. and Furusawa *et al*. reporting decrease survival fractions of Chinese hamster V79 cells at increased carbon-ion irradiation dosages^23,24^.

**Figure 2:**
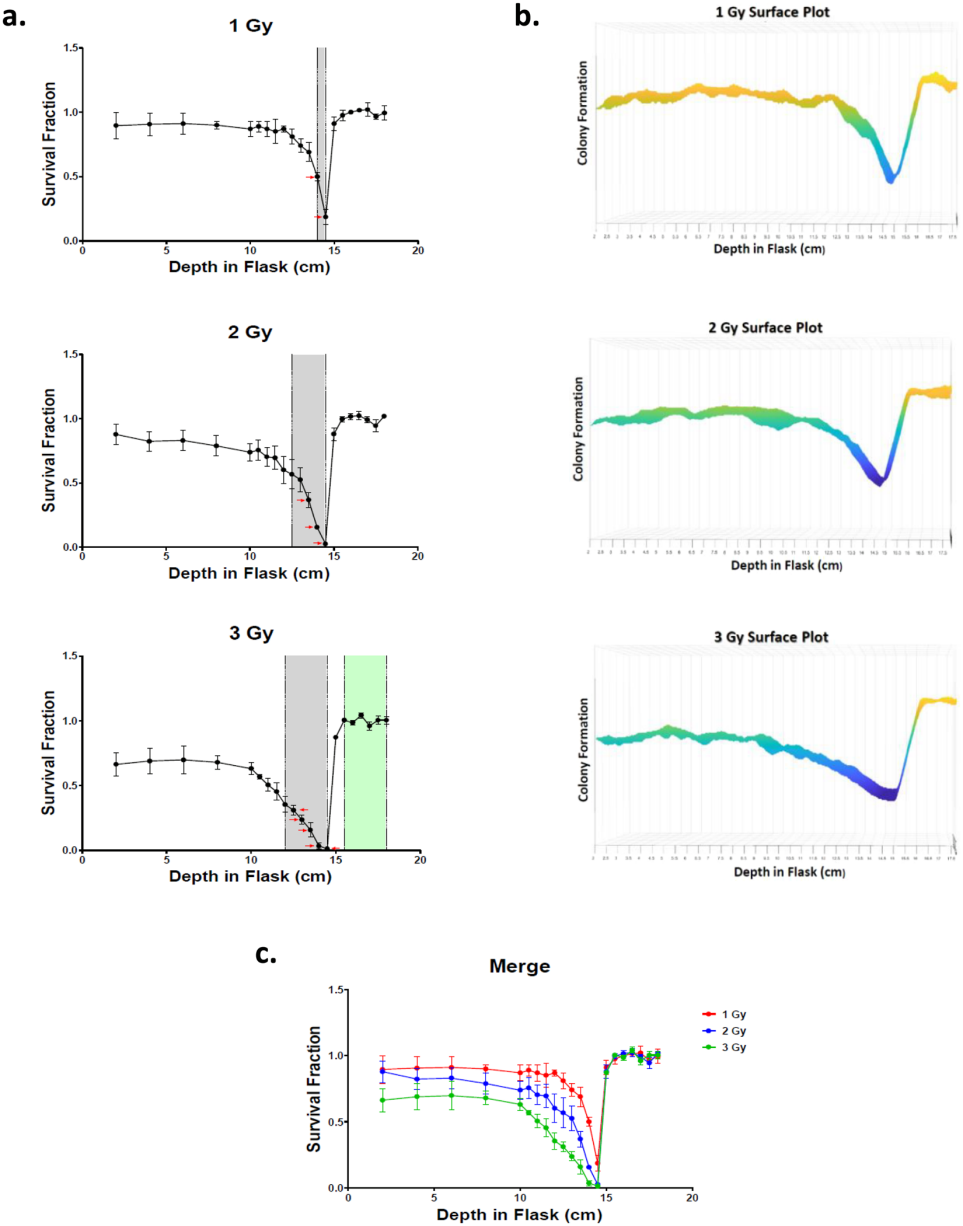
Cell survival vs depth following irradiation. (**a**) Survival fraction vs depth following treatment of 1, 2 or 3 Gy initial dosage. Areas highlighted in gray represent significant decrease (*P* < *0*.*05*), red arrows represent significant decrease (*P* < *0*.*0001*), areas highlighted in green represent significant increase (*P* < *0*.*05*), Dunnett’s Multiple Comparison Test. (**b**) Surface plot representation of colony formation at increasing depths for each experimental initial carbon-ion dosage. Surface plots were generated via MATLAB^TM^ software. The surface plot displays the relative density of cell colonies as a function of flask depth and flask width; higher densities yielded higher arbitrary values on the Z-axis and vice versa. The color scheme used was standardized among all plots. The order of the color scheme from maximum cell colony density to minimum cell colony density (few or no cells present) is as follows: yellow, orange, green, blue, and purple, respectively. (**c**) Merge of all cell survival curves. Error bars indicate standard errors of the means from a minimum of three independent experiments per each one of our initial dosages.

Flasks treated with 1 Gy initial irradiation portrayed significant decrease in survival fractions at depths of 14.0 cm and 14.5 cm (*P* < *0*.*0001*) (Fig. 2a). Treatment with 2 Gy initial irradiation portrayed significant decrease in survival fractions at depths between 12.5 and 14.5 cm (*P* < *0*.*05*) with the most significant decrease occurring between the depths of 13.5 and 14.5 cm (P < 0.0001). Treatment with 3 Gy initial irradiation portrayed a significant decrease in survival fractions between the depths of 12.0 and 14.5 cm (P < 0.05), with the most significant decrease being between 12.5 and 14.5 cm (P < 0.0001). Treatment at this initial dosage also demonstrated significant increases in survival fractions for the depths between 15.5 and 18.0 cm (*P* < *0*.*0001*) (Fig. 2a,c), which may have been due to observed lower survival fractions at beam entry with higher initial dosages. Moreover, under all of initial radiation dosages survival fractions were fairly similar for the depths from 15.0 cm and beyond, consistent with the carbon-ion beam having a sharp drop off of dosage following its Bragg peak and indicating that dosages at this depth and beyond were not enough to be considered lethal (Fig. 2c).

To ensure that our chosen evaluation depths of our previous cell survival assays depicted accurate survival fractions, a surface plot was generated via MATLAB^TM^ software for each of our cell survival experimental initial dosages (Fig. 2b). This method allowed for the evaluation of colony formation at all depths but could not distinguish between surviving colonies vs non-surviving colonies, i.e. if the colony contained >50 cells. However, these results portrayed a clear range in which there was a rapid decrease in colony formation. The depth possessing the least number of colonies was at 14.5 cm and following this depth there was a sharp increase in colony formation under all initial dosage conditions. Therefore, the consistencies between our surface plots and our previous survival assay results supported that our chosen evaluation depths were an accurate description of survival fraction changes.

**Figure 3:**
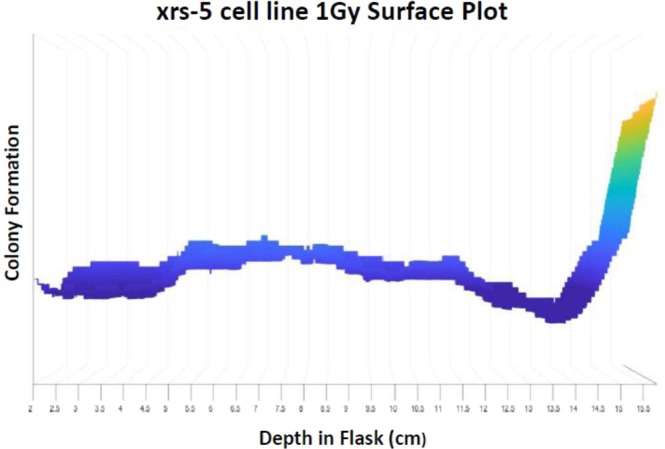
Colony formation vs depth of a radiosensitive cell line. The DNA double-strand break repair-defective cell line, xrs-5, demonstrates a dramatic decrease of colony formation from carbon-ion beam entry up to the biological Bragg peak depth.

Furthermore, with 1 Gy initial carbon-ion beam dosage to the radiosensitive cell line, xrs-5, we observed a dramatic decrease in colony formation from the initial evaluation depth up to the observed biological Bragg peak (Fig. 3). The xrs-5 cell line is a DNA double-strand break repair-defective cell line derived from the CHO-K1 cell line^25^. We utilized this cell line as xrs mutant cells have demonstrated to be one of the most radiosensitive cell lines so far isolated in mammalian cells^26,27^. This observed result in combination with our previous results indicated that DNA damage was induced from initial entry of the carbon-ion beam to the Bragg peak depth. However, cells possessing proper DNA repair mechanisms are more capable of overcoming the damage up to the proximal end of our observed biological lethal dose range. This is consistent with a previous study by W.K. Weyrather *et al*. in which they reported dramatic decreases in survival fractions of xrs5 cells at far lower doses than with CHO wild type cells^22^.

**Figure 4:**
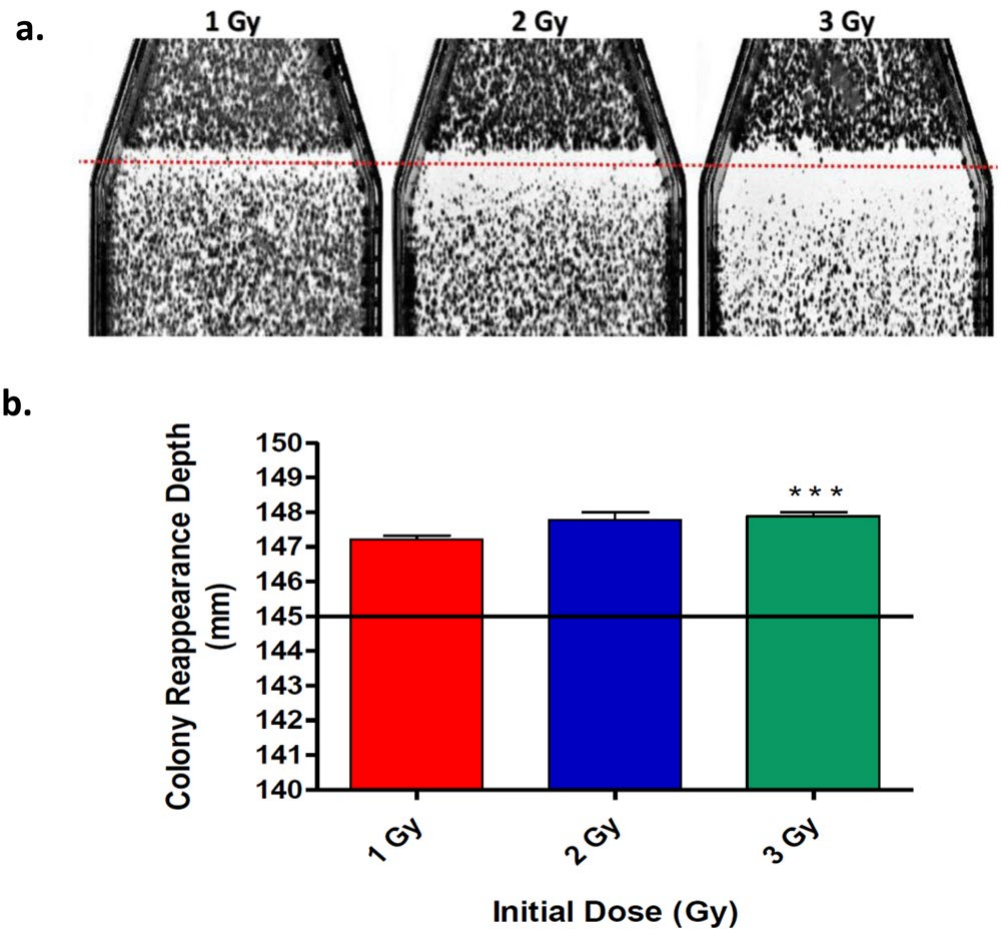
Reappearance of colony formation following biological Bragg peak. (**a**) Close up image of flasks in Fig. 1c following irradiation of carbon-ion beam at increasing dosages displaying the increased lethal dose range with an increased initial dosage. Dotted red line represents biological Bragg peak depth at 14.5 cm. (**b**) Colony reformation under all initial dosage treatments between depths of 14.7–14.8 cm. Increase of initial dosage led to significant increase in depth of colony reappearance (*P* = *0*.*0496*). Colony reappearance depth of 3 Gy was significantly increased as compared to 1 Gy (*P* < *0*.*05*), Dunnett’s Multiple Comparison Test. Straight line representative of our biological Bragg peak. Error bars indicate standard errors of the means from as many as three independent experiments per each initial dosage.

To determine the depth at which the dosage returned to a biologically non-lethal dose following our biological Bragg peak at 14.5 cm, we measured the depth at which there was colony reformation under each of our experimental initial dosages. This depth was representative as the distal end of our biological lethal dose range (Fig. 4a). Under all experimental initial dosages there was reappearance of colony formation observed around the depth of 14.7 ± 0.0357 cm. Colonies were observed for initial dosages of 1 Gy, 2 Gy and 3 Gy at the depths in the flask of 147.22 ± 0.4 mm, 147.77 ± 0.4 mm and 147.89 ± 0.3 mm, respectively. These results indicated that as the initial dosage increased from 1 Gy to 3 Gy the depth of colony formation significantly increased as well (*P* < *0*.*05*) (Fig. 4b).

### Micronuclei formation vs depth in the flask

**Figure 5:**
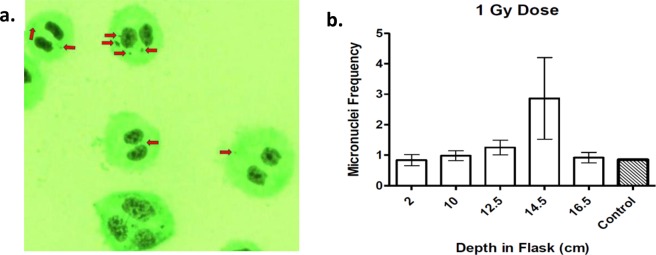
Micronuclei frequency at increasing depths in T-175 flasks. (**a**) Example of evaluated micronuclei formation. Red arrows indicate micronuclei evaluated from binucleated cells. (**b**) Micronuclei frequency observed to be highest at depth of 14.5 cm. Error bars indicate standard errors of the means from as many as three independent irradiation experiments, as well as, for our control.

Micronuclei (MN) were quantitatively evaluated to address carbon-ion beam irradiation-induced genotoxicity. CHO cell cultured T-175 flasks were subjected to an initial dose of 1 Gy carbon-ion beam irradiation entering from the bottom of the flask. The cytokinesis-block micronucleus assay (CBMN) was utilized, as it has been shown to be the procedure of choice to quantitatively evaluate micronuclei formation^28,29^. CBMN assays were conducted in Greiner T-175 cell culture flasks, following a 1-day incubation period of CHO cells irradiated with 290 MeV/n carbon-ions at an initial dosage of 1 Gy. The formation of micronuclei per 150 binucleated cells was quantified at increasing depths in the flask. Evaluated micronuclei are exemplified in Fig. 5a. We chose the depths to evaluate micronuclei formation at 2.0  and 10.0 cm, as there was a relatively small decline in colony formation between these depths in our previous 1 Gy initial dosage survival assay. The depths at 12.5  and 14.5 cm were chosen as we observed a steep decline in colony formation starting at 12.5 cm and ending at 14.5 cm. Our final chosen evaluated depth was at 16.5 cm, as this depth was observed to possess the highest number of colonies.

Our results portrayed that micronuclei formation increased in correspondence with an increased depth in the flask up to the evaluated depth of 14.5 cm. As compared to 14.5 cm, the depth of 16.5 cm displayed a significant decrease in micronuclei formation (*P* < *0*.*05*), with mean values of micronuclei per 150 binucleated cells of 2.865 ± 2.3 to 0.919 ± 0.30, respectively. However, micronuclei formation at any of our evaluated depths was not significant as compared to our control in which cells were not treated with irradiation (*P* = *0*.*2088*). It is important to note that micronuclei formation at the depth of 16.5 cm fell between micronuclei formation at the depths of 2.0 cm and 10.0 cm, with mean values of micronuclei per 150 binucleated cells of 0.837 ± 0.32 and 0.981 ± 0.28, respectively, and 0.919 ± 0.30 for 16.5 cm (Fig. 5b).

## Discussion

The main goal behind radiotherapy is to deprive cancer cells of being able to complete cell division, therefore the endpoint of cell survival is commonly used for *in-vitro* experiments^30^. Our new method for addressing cell survival has allowed us to express the survival fraction at increasing depths in a single system following carbon-ion beam irradiation, in which the classical cell survival method could not. This method was important for more accurately determining the range of depths at which the dosages could be considered biologically lethal by displaying significant losses in cellular clonogenicity in our T-175 flasks.

To further check if our evaluated depths depicted an accurate representation of survival fraction throughout our flasks we generated a surface plot for each of our experimental initial dosages (Fig. 2b). The advantage of this method was that it allowed us to observe colony formation at all depths in our flask to ensure that there were no abnormal anomalies indicated outside of our evaluated depths. However, the disadvantage of this approach was that it could not decipher between surviving colonies vs non-surviving colonies, i.e. if the colony contained >50 cells or not. Although, the results utilizing this technique were observed to be relatively consistent with our cell survival results, therefore supporting the accuracy of our chosen evaluated depths used for our cell survival assays.

In this study we observed few to no colony formation at the depth of 14.5 cm in our T-175 flasks, under all tested irradiation initial dosages (Figs 1c and 2c). Therefore, we determined this depth to be our biological Bragg peak as it was consistent with our Fricke gel indicating the highest observed dose values ranging between the depths of 13.8 and 14.5 cm (Fig. 1a). Furthermore, we observed a clear trend of decreasing survival fraction and increasing micronuclei formation as the depth increased up to the biological Bragg peak at 14.5 cm (Fig. 2a). However, our results were consistent with the carbon-ion displaying a sharp high dose Bragg peak^31^. Our CBMN assay results portrayed a relatively small increase in MN formation between the depths of 2.0 and 10.0 cm with a higher increase in MN formation at 12.5 cm and the most notable increase occurring at the biological Bragg peak following a 1 Gy treatment dose (Fig. 5a). Moreover, at each increase of treatment dose the range of significant decrease in survival fractions increased as well (*P* < *0*.*0001*), for the depths ranging between 14.0 and 14.5 cm, 13.5 and 14.5 cm and 12.5 and 14.5 cm for 1, 2 and 3 Gy, respectively (Fig. 2a). These results suggest that as the initial dosage is increased the range of lethal dose distribution prior to the Bragg peak also increases.

Beyond our biological Bragg peak we expected the depth of colony reformation to remain consistent regardless of initial dosage, as previous studies have indicated there should be a steep drop-off of energy deposition^9,10^. However, our results portrayed a significant increase in the depth of reappearance of colony formation following the biological Bragg peak, as the initial dosage increased from 1 Gy to 3 Gy, this depth increased from 147.22 ± 0.4 mm to 147.89 ± 0.3 mm, respectively (*P* < *0*.*05*) (Fig. 4b). In addition, we observed a higher number of MN at the depth of 16.5 cm as compared to the depth of 2.0 cm. This result conflicted with our cell survival assay depicting the highest survival fraction at the depth of 16.5 cm, indicating there should have been less MN at 16.5 cm than at 2.0 cm. However, it is important to note that our control indicated that CHO cells possessed a relatively high level of background MN formation as the initial evaluated depth of 2.0 cm portrayed similar MN formation as with our control (Fig. 5a). Taken together, these results suggest that the carbon-ion fragmentation tail is capable of inducing cell death and genotoxicity. Therefore, the carbon-ion lethal dose range may extend beyond the biological Bragg peak depth as well.

It is of clinical importance to repeat these experiments utilizing the clinically relevant spread-out Bragg peak (SOBP) 290 MeV/n carbon-ion irradiation technique to address if the SOBP technique produces cellular lethality to the same degree as the monoenergetic carbon-ion beam, as well as, if this technique demonstrates the same nuclear fragmentation effects as observed in this study. If it is found that the SOBP technique is consistent with our observed results utilizing the monoenergetic carbon-ion beam, this would require modifications in the dose calculation of therapy for physicians to address the nuclear fragmentation and avoid healthy tissue damage beyond the target tumor tissue. Furthermore, repeating these experiments with a proton beam will help specify the degree in which carbon-ion beams provide a more precise distribution of biological lethal dosages, thus providing more support for the expansion of these facilities over the more common proton facilities used today.

A limitation of this study consisted of being limited to cell lines whose colonies were not greater than 0.5 cm in diameter for our survival assay variation technique, in order to not incorporate a colony at more than one depth. Therefore, we used the CHO cell line, as no colonies were observed to be greater than this. Another limitation involves the lack of a way to accurately depict the exact dose distribution throughout our flask. Currently available methods for this calculation have demonstrated to be unreliable for highly heterogenous systems, making them unreliable for our technique utilizing live cells in media^32^.

## Materials and Methods

### Irradiation conditions

Carbon-ions were accelerated to 290 MeV/nucleon using the Heavy Ion Medical Accelerator in Chiba (HIMAC) synchrotron. Dose rates for the survival assays for carbon-ions were set at 1 Gy/min. Monoenergetic 290 MeV/nucleon carbon-ions have a LET value of 13 keV/μm on entrance. Irradiations were carried out at room temperature.

### Fricke Gel Dosimeter

Ferrous xylenol gel was made in a T-175 cell culture flask containing: 4% (wt) gelatin 300 Bloom from porcine skin (Sigma, St Louis, MO, Type Ag 2500), 50 mM sulfuric acid, 1 mM ferrous ammonium sulfate hexahydrate (Sigma), 0.1 mM xylenol orange (Sigma), and 96% Milli-Q water. After gel solidified, flask was irradiated at a dosage of 20 Gy monoenergetic 290 MeV/n carbon-ion beam. Carbon-ion beam entered from bottom (non-capped end), as described in the irradiation procedure and cell survival assays. 30 min following irradiation, flask was scanned to capture image.

### Cell culture

Chinese hamster ovary (CHO) cells and xrs5 cells were kindly supplied by Dr. Joel Bedford (Colorado State University, Fort Collins, CO). Cells were grown and maintained in α-MEM (Invitrogen, Carlsbad, CA) supplemented with 10% heat inactivated fetal bovine serum (Sigma), supplemented with antibiotics and antimycotics at 37 °C in incubators at 5% CO_2_ and 100% humidity. Doubling times were approximately 12 hours for this cell line.

### Irradiation procedure for cell survival assays

**Figure 6:**
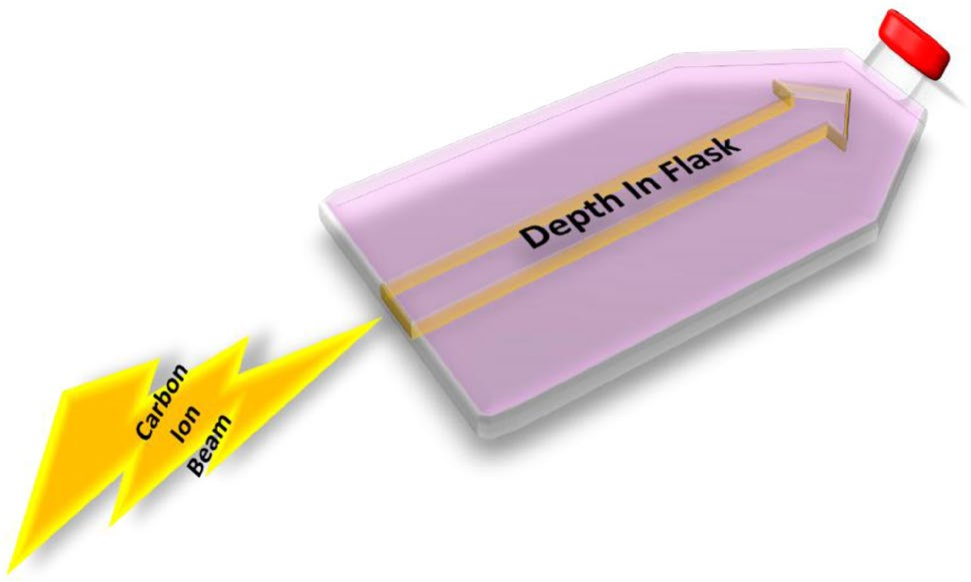
Illistration of carbon-ion beam entry. Prior to irradiation, cell culture flasks were placed upright with the capped end opposite to the beam source. Flasks were irradiated independently from each other and beam entry site, depected by lightnight bolt, was at the bottom of the flask (non-capped end).

Cultured cells were trypsinized and re-suspended into growth medium. Once re-suspended, 50 mL of media containing 30,000 cells were placed into their appropriately labeled T-175 cell culture flask approximately 1 hour prior to irradiation and attachment was confirmed. All flasks were irradiated independently with a dosage of either 1, 2 or 3 Gy. For all flasks, the beam entry point was at the bottom of the flask (non-capped end) (Fig. 6). Immediately following irradiation, all cells were incubated for a period of 7 days for colony formation. After this culturing period, each culture flask was then washed with 0.9% NaCl, fixed in 100% ethanol and stained with 0.1% crystal violet.

### Survival fraction calculation for cell survival assays

**Figure 7:**
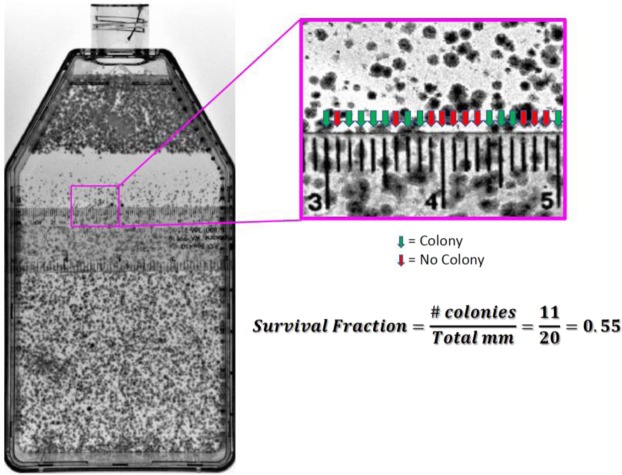
Example scoring procedure for calculating survival fraction. At each specified depth, surviving colony or no colony was addressed for every millimeter across the widths of the flask. Surviving colony was defined as a colony containing >50 cells. Survival fraction was equal to the number of surviving colonies over the total width of flask (mm).

Survival fraction was obtained at depths of every 2 cm from 2 to 10 cm and every 0.5 cm from 10  to 18 cm from carbon-ion beam entry at the bottom of the flask (non-capped end). To quantify the survival fraction at each of our evaluated depths, they were scored for every millimeter along the width of the flask either possessing a surviving colony, defined as a colony containing >50 cells, or not possessing a surviving colony and the average value was calculated (Fig. 7). This approach was repeated for a minimum of three independent experiments per each one of our initial dosages of 1, 2 or 3 Gy.

### Computer generated surface plots of colony formation

Survival assay flasks were imaged with the BIO-RAD ChemiDoc chemiluminescent imager (BIO-RAD, Hercules, CA) via ImageLab^TM^ 2.0.1 software (BIO-RAD) under white trans illumination utilizing a standard emission filter. These images were binarized and converted into a surface plot via MATLAB^TM^ software (MATHWORKS, Natick, MA). Pixel differences addressed colony distribution incorporating all colonies between the widths of 3 to 8 cm and depths of 2 to 17.5 cm.

### Irradiation procedure and Cytokinesis-Block Micronucleus Assay (CBMN)

100,000 cells were placed into their appropriately labeled T-175 cell culture flask approximately 1 hour prior to 1 Gy of irradiation. Immediately following irradiation, cells were treated with 250 μL of cytochalasin B (1 mg/mL) for a final concentration of 5 μg/mL and incubated for a period of 1 day. After this incubation period, cells were fixed by: discarding the media, washing with PBS, followed by treatment of 20 mL 75 mM KCl for 10 seconds and then discarded, followed by treatment with 20 mL solution of Acidic Acid and Methanol (1:1) for 10 seconds and then discarded to make flasks dry. After fixation, cells were stained for 5 minutes with a solution composed of 30 mL Gurr (Gibco, Waltham, MA) with 1.5 mL Giemsa (Gibco). A minimum of three independent CBMN assay experiments were carried out for our irradiated samples, as well as, for our control. Micronuclei frequency was determined by the number of micronuclei divided by the number of binucleated cells. Approximately 150 binucleated cells in irradiated samples were evaluated at each depth of 2.0, 10.0, 12.5, 14.5, and 16.5 cm. Since our control samples were independent of depth, approximately 150 binucleated cells were evaluated regardless of depth.

### Statistical analysis

All experimental data were analyzed via Prism 5^TM^ software (GraphPad, La Jolla, CA). One-way analysis of variance (ANOVA) and Dunnett’s multiple comparison test was conducted for statistical significance. P-values of <0.05 were considered to indicate differences that were statistically significant.

## Conclusions

In conclusion, our approach to the *in-vitro* cell survival method and CBMN assays allows for a more biologically representative assessment of the range in which carbon-ion irradiation induces cell death. The results of this study suggest that monoenergetic 290 MeV/n carbon-ion beams portrayed a biological Bragg peak at the depth of 14.5 cm in our flask. Furthermore, that there is a range in which the carbon-ion beam distributed lethal dosage both prior to and following its Bragg peak depth, which increased with an increased initial dosage. Moreover, our results suggested that nuclear fragmentation of the carbon-ion beam may be responsible for cell death and genotoxicity beyond the carbon-ion beam Bragg peak. Defining this biological lethal dosage range, will aid physicians in accurately targeting tumor tissues while limiting cell death in the surrounding normal tissues.
